# Knowledge, attitudes, and practices among oncologists regarding the implementation of DRGs payment system: a cross-sectional study in Beijing

**DOI:** 10.3389/fpubh.2024.1453962

**Published:** 2024-12-23

**Authors:** Changdan Xu, Hong Zhang, Shiquan Yin

**Affiliations:** Department of Medical Record, National Cancer Center/National Clinical Research Center for Cancer/Cancer Hospital, Chinese Academy of Medical Sciences and Peking Union Medical College, Beijing, China

**Keywords:** knowledge, attitude and practice, KAP survey, validity, DRGs, Diagnosis Related Groups

## Abstract

**Background:**

The KAP survey evaluates health-related knowledge, attitudes, and practices through a structured questionnaire. By collecting qualitative and quantitative data, it measures the current situation, tests hypotheses, and provides insights for enhancing health behaviors and education. In 2019, the National Health Security Administration (NHSA) initiated DRG payment reforms. This study aims to improve the quality of health insurance and policy implementation by assessing physicians' knowledge, attitudes, and practices regarding the DRG system.

**Method:**

This study was a cross-sectional study designed with a questionnaire through simple random sampling method, and respondents were the doctors in the clinical departments of the sampled hospitals. The questionnaire included basic information, knowledge about DRGs, attitude toward DRGs and practice of implementation. Data were analyzed using descriptive statistical analysis, correlation, path analysis and generalized linear model.

**Result:**

A total of 210 questionnaires were included. The majority of respondents aware that their healthcare organizations had already begun to implement the policy. With a mean score of 7.67 for knowledge, respondents basically had a good level of knowledge of DRGs. The mean attitude score of the respondents was 30.20, which was lower than the “positive attitude” criterion, and their main concerns were about matters other than treatment. Knowledge scores were significantly correlated with attitude scores (*P* < 0.001), whereas attitude scores were not associated with practice scores. Path analysis and generalized linear modeling indicate that knowledge effectively influences attitudes, whereas attitudes do not have an apparent impact on practice.

**Conclusion:**

Oncologists' understanding of DRGs needs to be improved, and their knowledge and attitudes have not yet translated into demonstrable positive practice behaviors. This gap underscores the need for knowledge training and effective incentives.

## 1 What is KAP survey

Knowledge, attitude, and practice (KAP) surveys were initially started in the 1950s in the fields of family planning and population research (1). Also known as knowledge, attitude, behavior, and practice surveys, these are now widely accepted for the investigation of health-related behaviors and health-seeking practices (2). The aim of the KAP survey is to elicit what is known (knowledge), believed (attitude), and done (practiced) in the context of the topic of interest. Information is collected using semistructured or (more usually) structured questionnaires that are self-administered or administered by interviewers; both qualitative and quantitative data are collected (3). KAP survey can be useful to measure the extent of a known situation/condition, confirm or disprove a hypothesis, and provide new horizons. It may be useful to enhance the knowledge, attitude, and practices of specific themes to identify what is known and done about various health-related subjects. KAP survey can be useful to establish the baseline reference value for use in future assessments/research and help measure the effectiveness of health education activities' ability to change health-related behaviors. The literature on the application of KAP method in public health, disease awareness, and behavioral intervention is relatively common (4–6), and the application of KAP method in medical decision-making behavior change is less (7, 8).

## 2 DRG payment system in China

China has successfully attained the goal of providing health insurance coverage to almost the entire population by developing a mixed health insurance system, which consists of Urban Employees Basic Medical Insurance (UEBMI), Urban Resident Basic Medical Insurance (URBMI), New Rural Cooperative Medical Scheme (NCMS), and supplementary Catastrophic Health Insurance (9).

In 2018, the Chinese government established the National Healthcare Security Administration (NHSA) to manage Basic Medical Insurance. In 2019, the National Healthcare Security Administration launched the DRG payment reform in 30 piloting cities across the country, using the CHS-DRG which comprises a total of 376 adjacent-DRG groups and 618 DRG subgroups (10).

In 2021, NHSA issued the “Three-year Action Plan for DRG/DIP Payment method reform,” requiring fully complete the task of DRG/DIP payment mode reform and promote the high-quality development of medical insurance. By the end of 2024, all coordinating regions in the country will carry out the reform of DRG/DIP payment methods, and pilot regions will start to consolidate the reform results. By the end of 2025, the DRG/DIP payment method will cover all eligible medical institutions carrying out inpatient services. In 2021, the National Medical Insurance Administration published a list of 39 DRG/DIP payment Demonstration sites, including provincial cities such as Beijing, Shanghai and Tianjin. Beijing is the city where the sample hospitals of this study are located.

Our research interest focuses on DRG payments system. Understanding physicians' knowledge, attitudes, and practices (KAP) and their perceptions of challenges, barriers, and facilitators toward Diagnosis Related Groups (DRG) payment system are vital in informing the improvement and implementation of successful policy delivery.

## 3 Methods

### 3.1 Questionnaire design

This research was formulated by reviewing relevant policy documents and literature, and referring to similar studies (7, 11, 12). A cross-sectional survey design was employed, with the respondents being physicians in the clinical departments of the sampled hospitals. A simple random sampling method was used, the sample size was calculated to include over 40% of the total staff. The overall standard deviation (σ) was estimated to be 0.95, with a permissible error (δ) of 0.15. The questionnaires were distributed using Questionnaire Star online platform. In this research, it is hypothesized that the level of doctors' DRG knowledge will affect their attitudes toward DRG payment, and the attitudes will in turn affect medical behaviors. Based on the above hypothesis, the questionnaire was divided into 4 parts, including basic personal information, knowledge about DRGs, attitude toward DRGs, and practical implementation.

The DRGs knowledge questions for oncologists were formed through literature review and consultation with medical insurance experts. Reverse questions were set on the first knowledge question and the second practice question. Before the formal survey was started, a pre-survey was first conducted among the relevant medical staff to refine the questionnaire, ensuring its accuracy and that it effectively captured the study's objectives. Formal questionnaire survey was conducted from January to July 2024.

Knowledge scores were calculated by awarding 1 point for each correct answer, no points for incorrect or unclear answers, question 4, 7, 8 were reverse scoring, and a total score ranging from 0 to 10, with higher scores indicating greater knowledge of DRGs. The attitude section was scored on a 5-point Likert scale ranging from strongly agree (five points) to strongly disagree (one point), with a total attitude score ranging from 9 to 45 points (questions with reverse scoring are labeled in the Tables 2–4). The practice section was rated on a scale of 1–3, with a total score range of 6–18. With more than 70% (13, 14) of the total score being considered good knowledge, positive attitude, and positive practice.

### 3.2 Statistical analysis

Statistical analysis was performed using SPSS 26.0, descriptive statistical analysis was expressed as frequency (percentage) or mean and standard deviation (*M* ± SD) of the variables. S–W test was used to test for normality, Spearman's correlation was used to analyze the correlation between knowledge, attitude, and practice scores. Kruskal–Wallis test and chi-square test were used to analyze the differences of knowledge, attitude, and practice across independent variables. Path analysis of knowledge, attitude, and practice and generalized linear model (GLM) were performed using SPSSPRO.^1^

## 4 Result

### 4.1 Baseline characteristics

**Figure 1:**
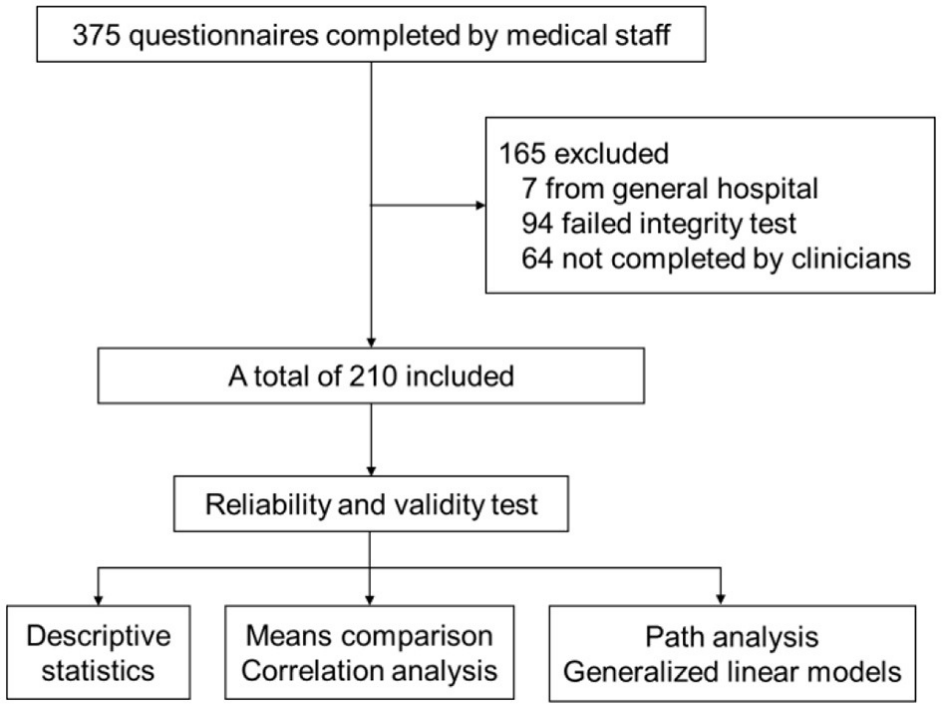
Research process.

A total of 375 completed questionnaires were collected in this study, seven from general hospitals, 94 failed the integrity test of reverse questions and 64 questionnaires that were not completed by clinicians were excluded, finally a total of 210 copies of questionnaires were included in the statistical analysis (Figure 1). The standardized Cronbach's α coefficient value of the questionnaire was 0.728, which was acceptable for reliability. The KMO test value is 0.706 and *P* < 0.05 for Bartlett's test indicates that the validity of the questionnaire is appropriate for factor analysis.

Subjects participated in the questionnaire survey were from one tertiary and two secondary oncology specialty hospital in Beijing, a city which officially launched the actual payment system based on Diagnosis Related Groups (DRGs) in March 2022. The professional backgrounds of the respondents were mainly concentrated in internal medicine (50.5%), surgery (23.8%), radiation therapy (10.0%) and Integrated medicine (8.1%). More than half of the respondents (58.6%) have more than 10 years of relevant work experience (Table 1).

**Table 1:** Basic information of participants in the survey.

**Characteristics, *n* (%)**	**Frequency**
**Department**	
Integrated medicine	17 (8.1)
Internal medicine	106 (50.5)
Surgery	50 (23.8)
Radiotherapy	21 (10.0)
Endoscopy	7 (3.3)
Traditional Chinese medicine	3 (1.4)
Intervention	1 (0.5)
Other	5 (2.4)
**Gender**	
Male	99 (47.1)
Female	111 (52.9)
**Age**	
20–29	32 (15.2)
30–39	79 (37.6)
40–49	71 (33.8)
50–59	24 (11.4)
Over 60	4 (1.9)
**Professional title**	
Senior	65 (31.0)
Intermediate	95 (45.2)
Junior	43 (20.5)
Other	7 (3.3)
**Years of experience**	
10 years or more	123 (58.6)
5–10 years	34 (16.2)
3–5 years	28 (13.3)
< 3 years	25 (11.9)
**Rank of the working hospital**	
Tertiary	87 (41.4)
Secondary	123 (58.6)
**Whether your hospital has begun to implement the actual**	
**payment of health insurance based on DRGs**	
Yes	188 (89.5)
No	15 (7.1)
Currently unknown	7 (3.3)
**Whether your hospital has incorporated DRGs-related**	
**indicators into its performance appraisal index system**	
Yes	160 (76.2)
No	21 (10.0)
Currently unknown	29 (13.8)

The results showed the degree of acceptance of the implementation of the actual payment policy of health insurance based on Disease Diagnosis-Related Groups (DRGs) by healthcare organizations, with the vast majority of respondents (188, 89.5%) indicating that their institutions have already begun to implement the policy, and only a small number (22, 10.5%) deny or not sure about it. Regarding the question of whether DRGs-related indicators have been incorporated into the hospital's performance appraisal index system, most respondents answered in the affirmative (160, 76.2%; Table 1).

### 4.2 Clinicians' knowledge of the DRG payment system

The average basic knowledge score of survey respondents was 7.67 (*M* = 7.67, SD = 2.043), and 127 (60.5%) had a well-developed level of knowledge of DRGs. Respondents were relatively knowledgeable about basic DRG grouping logic, but there was a common misunderstanding on one question, where respondents believed that the reimbursement rate for a UEBMI patient's hospitalization could possibly affect their DRGs enrollment for the current discharge, with only 51.9% of survey respondents correctly answering that the statement was incorrect (Table 2). 144 (68.6%) of the respondents indicated that it is wrong that any cases treated with expensive and high-priced medications can be paid separately on a waiver basis, but one-third considered the statement to be correct or not sure.

**Table 2:** Distribution of knowledge among the participants.

**Knowledge items, *n* (%)**	**Correct**	**Incorrect**	**Don't know**
DRGs grouping is a method of casemixing routinely cases	188 (89.5)	7 (3.3)	15 (7.1)
The DRGs grouping focuses on the dimensions of clinical process consistency and resource consumption similarity	188 (89.5)	6 (2.9)	16 (7.6)
Cases in the same DRGs subgroup require similar clinical procedures and similar resource consumption	176 (83.8)	20 (9.5)	14 (6.7)
The reimbursement rate for a UEBMI patient's hospitalization affects his or her DRGs enrollment for the current hospital discharge (reverse scoring)	64 (30.5)	109 (51.9)	37 (17.6)
The primary diagnosis and primary surgical operation for which the patient was hospitalized was the determining factor in determining the group of DRGs	198 (94.3)	3 (1.4)	9 (4.3)
DRGs groups can be used to compare efficiency and quality of medical care among different hospitals	150 (71.4)	33 (15.7)	27 (12.9)
Birth weight is necessary for DRGs grouping (reverse scoring)	22 (10.5)	127 (60.5)	61 (29.0)
Cases treated with costly, high-priced medicines are separately paid as special cases (reverse scoring)	26 (12.4)	144 (68.6)	40 (19.0)
Compared with the previous period, payment by DRGs is conducive to controlling the increase of medical expenses	161 (76.7)	17 (8.1)	32 (15.2)
The implementation of actual payment by DRGs by the Beijing Municipal Health Insurance Bureau is on March 2022	169 (80.5)	8 (3.8)	33 (15.7)

### 4.3 Clinicians' attitudes toward the DRG payment system

The attitude score average among respondents was 30.20. Only 40.5% of the doctors said that “the medical staff can be more effective in helping the patients under the DRGs payment system,” though their main concerns extend beyond treatment-related issues. A significant majority of respondents (88.1%) said that “grouping by DRGs will force clinicians to pay more attention to the cost of patient care.” 81.0% participants expressed that clinicians should devote more effort to the realization of clinical pathways. It is noteworthy that many (74.3%) of the respondents indicated that “my hospital has performed a good cost measurement for the payment system based on DRGs” (Table 3). However, the implementation of DRGs may not be conducive to the use of medically innovative technologies, which has a negative impact on diagnostic and therapeutic activities.

**Table 3:** Distribution of attitudes among the participants.

**Characteristics, *n* (%)**	**Strongly agree**	**Agree**	**Neutral**	**Disagree**	**Strongly disagree**
Compared to the previous period, medical staff are able to help patients more effectively after payment by DRGs	37 (17.6)	48 (22.9)	63 (30.0)	50 (23.8)	12 (5.7)
I have had to deal with more administrative issues in order to adapt to the implementation of payment by DRGs	77 (36.7)	87 (41.4)	34 (16.2)	9 (4.3)	3 (1.4)
Payment by DRGs system will possibly increase my personal income	3 (1.4)	10 (4.8)	101 (48.1)	56 (26.7)	40 (19.1)
Grouping by DRGs will force clinicians to focus more on the cost of patient therapy	115 (54.8)	70 (33.3)	19 (9.0)	5 (2.4)	1 (0.5)
Payment by DRGs groups hospitals to declare a faster return of health insurance funds	13 (6.2)	27 (12.9)	158 (75.2)	6 (2.9)	6 (2.9)
Clinicians should invest more effort in achieving clinical pathways	73 (34.8)	97 (46.2)	24 (11.4)	13 (6.2)	3 (1.4)
The hospital I work at has made good cost measurements for the DRGs-based payment system	51 (24.3)	105 (50.0)	52 (24.8)	1 (0.5)	1 (0.5)
Payment system based on DRGs will probably restrain physicians' career development (reverse scoring)	32 (15.2)	64 (30.5)	77 (36.7)	34 (16.2)	3 (1.4)
Payment system based on DRGs is not favorable to the use of medical innovations (reverse scoring)	56 (26.7)	74 (35.2)	53 (25.2)	17 (8.1)	10 (4.8)

### 4.4 Practice

Practice was mainly reflecting on the consumption of medical resource and the standardization of the medical process. The average score for practice was 16.34 (*M* = 16.34, SD = 1.624). The most commonly performed practice by clinicians was “matching the primary operation or procedure with the primary diagnosis of the current hospitalization” (93.3%), followed by other diagnoses reported on the first page as a serious complication in the medical record (89.0%). 88.1% of the respondents' primary diagnosis reported at home page was the diagnosis of the disease that most consumed the resources of the current hospitalization (Table 4).

**Table 4:** Distribution of practice among the participants.

**Practice items, *n* (%)**	**Yes**	**No**	**Moderately**
The primary diagnosis reported on the home page should be the diagnosis of the disease that consumes the most resources in this hospitalization	185 (88.1)	15 (7.1)	10 (4.8)
Clinical pathways can only be applied to patients hospitalized for chronic treatment (reverse scoring)	7 (3.3)	147 (70.0)	56 (26.7)
The primary procedure or operation is written to match the primary diagnosis of this hospitalization	196 (93.3)	2 (1.0)	12 (5.7)
The primary diagnosis of a discharged case determines its classification in an MDC group, while other diagnoses determine its classification in a specific DRG group	121 (57.6)	52 (24.8)	37 (17.6)
ICD-10 is used for primary diagnosis and other diagnostic codes	168 (80.0)	5 (2.4)	37 (17.6)
Other diagnosis reported as a serious comorbidity on the home page need not be reflected in the medical record (reverse scoring)	12 (5.7)	187 (89.0)	11 (5.2)

### 4.5 Correlation of knowledge, attitude and practice

**Figure 2:**
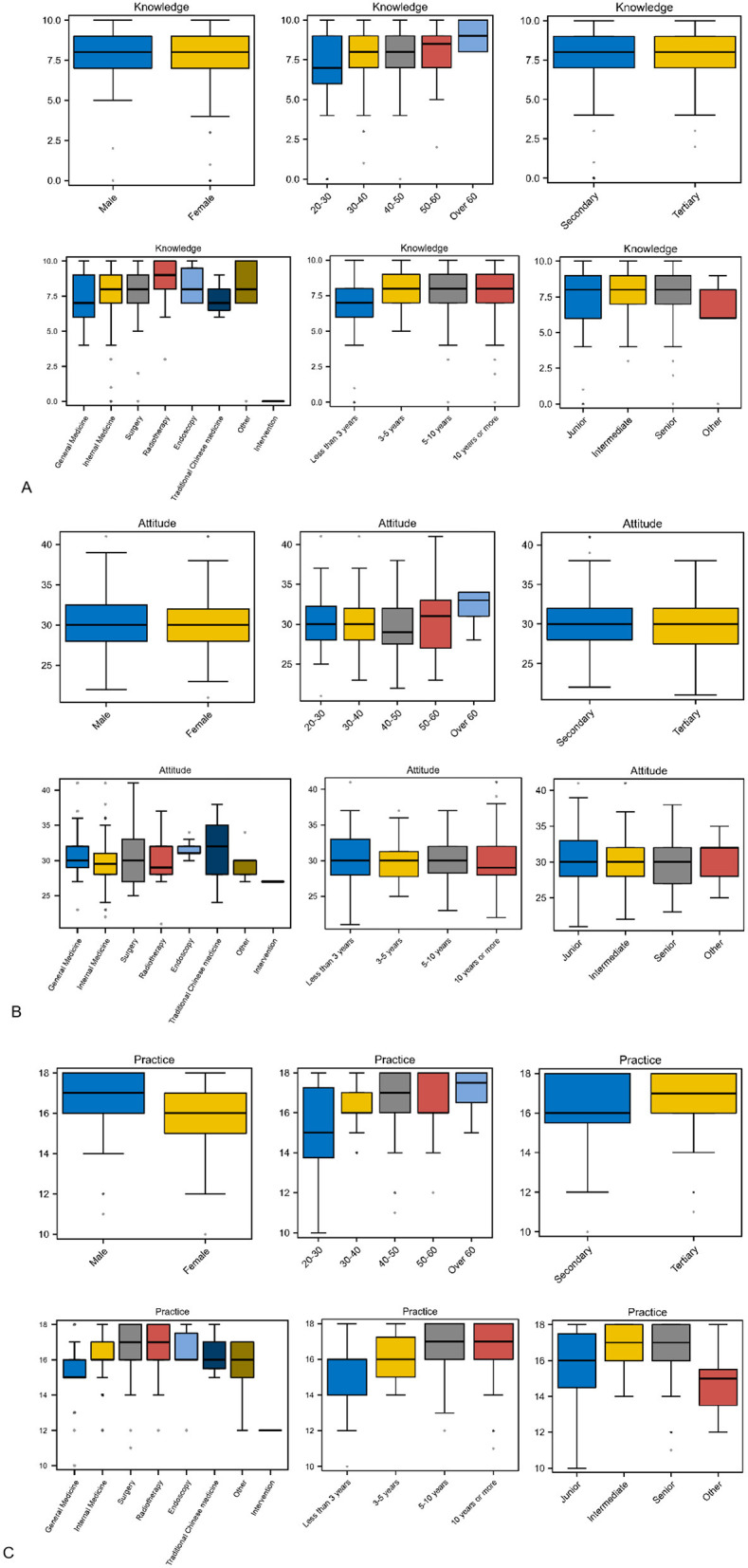
Box plots of total knowledge, attitude, and practice scores. **(A–C)** show the distribution of knowledge, attitude, and practice scores by gender, age, hospital level, department, years of experience, and years of title, respectively. Practice scores differed significantly by age, gender, title, department, and years of experience though there was no significant difference in knowledge and attitude scores.

There were no statistical differences in knowledge or attitude scores among physicians of different ages, genders, titles, years of experience, and departments (Figures 2A, B). However, practice scores varied significantly by age (*P* = 0.007), gender (*P* = 0.004), title (*P* = 0.003), department (*P* = 0.007), and years of experience (*P* = 0.016; Figure 2C). No significant differences in KAP scores were observed between doctors working in tertiary and secondary hospitals (*P* > 0.05). Chi-square analysis revealed that positive practice rates differed by age (*P* = 0.003), title (*P* = 0.02), and years of experience (*P* = 0.011).

**Figure 3:**
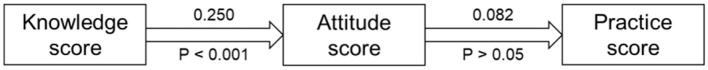
Model Paths. The path of knowledge effecting attitudes is valid with an impact coefficient of 0.250; the path from attitudes to practices is not significant.

Knowledge scores were positively correlated with attitude scores, with a spearman's correlation coefficient of 0.274 (*P* < 0.001), suggesting that better knowledge may be associated with more positive attitudes. However, there was no significant relationship between attitude and practice scores (*P* = 0.363). Path analysis further indicated that knowledge level had a direct impact on attitude, with an effect coefficient of 0.250 (*P* < 0.001), while attitude scores did not significantly influence practice scores (*P* > 0.05; Figure 3).

Subsequently, a generalized linear model was developed to examine the relationships between knowledge and attitude, as well as attitude and practice. The Omnibus test results indicated that both models were statistically significant (*P* = 0.002; *P* < 0.001). It is noted that the value of the model fit indices is higher than expected. In the model of knowledge and attitude (model 1), the coefficient for the statement “Payment by DRGs is conducive to controlling the increase of medical expenses” was positive and highly significant (*P* < 0.001), with a coefficient of 2.555 (Supplementary Table 1). The statements “Payment by DRGs system will possibly increase my personal income” and “Clinicians should invest more effort in achieving clinical pathways” were also highly significantly contributed in the model of attitude and practice (model 2), with partial coefficients are negative (Table 5, Supplementary Table 2).

**Table 5:** Effects of generalized linear models.

	**Model 1**	**Model 2**
Statistically significant factors	Compared with the previous period, payment by DRGs is conducive to controlling the increase of medical expenses (*P* < 0.001)	Payment by DRGs system will possibly increase my personal income (*P* < 0.001)
		Clinicians should invest more effort in achieving clinical pathways (*P* = 0.009)
AIC	1,126.057	801.687
BIC	1,166.222	928.877

## 5 Discussion

To the best of our knowledge, this is the first study to examine the cognition and practice of the DRGs payment system among a group of oncology specialists in China. In this study, it was discovered that 2 years after healthcare institutions had begun to implement the DRGs payment policy, oncology clinicians' knowledge of DRGs was still insufficient, with only 60.5% of which having good knowledge of DRGs. Although the level of knowledge affects physicians' attitudes to some extent, this effect does not convert into significant positive practice behaviors.

Medical institutions have accepted the diagnosis-related groups (DRGs)-based UEBMI payment policy to a large extent and have integrated DRGs-related indicators into their performance evaluation systems, but this has not produced the expected positive effects on the actual work of clinicians. Nearly half (40.5%) of physicians believe that the implementation of the DRGs payment policy will enable them to serve patients more effectively. The main concerns of clinicians were in terms of possibly under-treatment, potential negative impacts on the quality of care and patient safety, and detrimental to the development of new medical technologies. Similarly, a study by Fässler et al. (11) showed that physicians believed that the professional principles could not be applied to all situations, so that the patient-centered quality of service had declined, and the efficiency of the consultation and treatment had not been improved since the introduction of DRGs. A meta-analysis by Meng et al. (15) found that DRG-based payments may save costs by reducing length of stay but do little to reduce readmission rates. However, a study from Zhejiang, China, showed (16) that even during the COVID-19 pandemic, the DRG policy implemented in Wenzhou showed its positive effects, which were mainly in promoting the public general hospitals to improve their comprehensive capacity and effectively reducing the disparity in the cost efficiency of treating similar diseases.

In contrast to the assumptions of general KAP model, in our study it was not observed that knowledge and attitude have a positive impact on practice, which may be due to the fact that at the initial stage of DRG reform, doctors are not yet fully cognitively aware of the system. In general, when physicians are initially confronted with DRG, they are relatively passive and their diagnostic and therapeutic behaviors are constrained to a certain extent due to inertial resistance to the reform and a shallow understanding of DRG. As understanding deepens, there will be a gradual recognition of the scientific nature of the DRG system as a medical management tool, which will increase the acceptance of DRG. When doctors realize that the implementation of DRG has a certain impact on medical staff's diagnosis, treatment habits and workload, they will gradually develop a gaming mentality and change from passive to active, thus affecting diagnosis and treatment behaviors (17). Therefore, it is necessary to improve physicians' knowledge and depth of understanding through more systematic and comprehensive training, such as targeted training programs, incentive mechanisms, and to motivate physicians to transform their knowledge into positive behaviors in practice through system design and feedback mechanisms.

In addition, potential influences were identified through a generalized linear model, though the number was minor. Entries with positive impact coefficients that did not reach statistical significance may have a potentially positive impact and need to be further investigated by expanding the sample size, and for entries with both positive and negative impact coefficients or high *P*-values, there may be ambiguity in questioning or a lack of consensus in the perceptions of medical staff. The new technologies and drugs in the field of oncology are developing rapidly, although it is in line with the original intention of the DRGs system aimed at improving the quality and efficiency of medical care, there is a need to evolve a payment system that is both beneficial to the regulation of medical behavior and promotes the development of clinical oncology.

It is discovered that only the practice scores varied, for reasons that may be related to physicians' incomes. With the implementation of the DRG version 2.0 grouping program, the grouping will be more standardized. Cases that are not suitable to be paid according to the DRG standard due to long hospitalization time, high medical costs, use of new drugs and consuming new technologies, complex and critical illnesses, or multidisciplinary joint diagnosis and treatment, etc., medical institutions can independently declare special cases for single negotiation. Medical institutions are called upon not to use the DRG payment standard as a quota to assess medical staff or link it to performance allocation indicators. The adjustment is beneficial for medical staff to focus more on the patient's disease itself on the basis of standardized treatment, so as to better help the patient.^2^

It is noteworthy that our study demonstrated a generally improved treatment regularity under the DRGs payment system, which reflects the positive impact of the DRGs. Similar results were reported in a study by Kim et al. (18). Zhang et al. (8) showed that the DRGs payment system reduces over-treatment and improves the efficiency of the consultation and treatment. In comparison with previous studies (7, 17), the advantage of this study lies in the fact that we emphasized the impact of the standardization of homepage reporting on medical practice. The standardization of homepage reporting is not only directly related to the accuracy and integrity of diagnostic data, but is also a key factor in determining the accuracy of DRGs coding, and the correct recording of complications or comorbidities is a potentially beneficial option under the DRG payment policy (19), which further affects the compensation structure of the healthcare organization. With standardized homepage reporting, we are able to obtain essential data for evaluating the quality of care and patient safety, which provides an important basis for healthcare organizations to improve their services and enhance patient satisfaction and safety.

There are also limitations in our study, such as a questionnaire design that does not comprehensively cover all aspects of oncologists' practice of DRG, and questionnaire entries that were not further categorized for more in-depth analyses. Despite the statistical significance of the generalized linear model, there are limited factors affecting the dependent variable, which indicates that these variables contributed minimally to the actual impacts related to DRGs in the context of this data collection. More compatible medical behaviors with DRGs need to be explored. The content of the questionnaire needs to be adapted toward a more direct and distinguishing direction. To enhance the rigor and credibility of future research, it will be important to further optimize the questionnaire structure, conduct confirmatory factor analyses, and expand the sample size.

## 6 Conclusion

Oncology clinicians' understanding of DRGs is still inadequate, and their level of knowledge and attitudes have not yet been translated into demonstrable positive practice behaviors, which requires intensive knowledge training and implementing effective incentives.

## Data Availability

The raw data supporting the conclusions of this article will be made available by the authors, without undue reservation.

## Footnotes

^1^ https://www.spsspro.com/

^2^ https://www.nhsa.gov.cn/art/2024/7/23/art_104_13313.html
