# Supplementary material for: Knowledge, attitudes, and practices among oncologists regarding the implementation of DRGs payment system: a cross-sectional study in Beijing
Source: Front Public Health. 2024 Dec 23;12:1453962. doi: 10.3389/fpubh.2024.1453962 (PMC11700967; doi:10.3389/fpubh.2024.1453962)
Supplement: Supplementary file 1 [file Table_1.docx]

Supplementary table 1 Parameter estimates of knowledge effects on attitudes

| Parameters (Knowledge item) | Coefficient | Standard error | 95% Wald CI | P value |
| --- | --- | --- | --- | --- |
| Intercept | 27.251 | 1.110 | 25.076—29.426 | < 0.001 |
| 1. DRGs grouping is a method of casemixing routinely cases; =1 | -0.771 | 0.907 | -2.549—1.006 | 0.395 |
| 1. DRGs grouping is a method of casemixing routinely cases; =0 | 0 |  |  |  |
| 2. The DRGs grouping focuses on the dimensions of clinical process consistency and resource consumption similarity; =1 | -0.228 | 0.975 | -2.139—1.683 | 0.815 |
| 2. The DRGs grouping focuses on the dimensions of clinical process consistency and resource consumption similarity; =0 | 0 |  |  |  |
| 3. Cases in the same DRGs subgroup require similar clinical procedures and similar resource consumption; =1 | 0.476 | 0.788 | -1.068—2.021 | 0.545 |
| 3. Cases in the same DRGs subgroup require similar clinical procedures and similar resource consumption; =0 | 0 |  |  |  |
| 4. The reimbursement rate for a UEBMI patient's hospitalization affects his or her DRGs enrollment for the current hospital discharge; =1 | 0.295 | 0.495 | -0.674—1.264 | 0.550 |
| 4. The reimbursement rate for a UEBMI patient's hospitalization affects his or her DRGs enrollment for the current hospital discharge; =0 | 0 |  |  |  |
| 5. The primary diagnosis and primary surgical operation for which the patient was hospitalized was the determining factor in determining the group of DRGs; =1 | 0.685 | 1.168 | -1.604—2.974 | 0.558 |
| 5. The primary diagnosis and primary surgical operation for which the patient was hospitalized was the determining factor in determining the group of DRGs; =0 | 0 |  |  |  |
| 6. DRGs groups can be used to compare efficiency and quality of medical care among different hospitals; =1 | 0.148 | 0.561 | -0.952—1.247 | 0.792 |
| 6. DRGs groups can be used to compare efficiency and quality of medical care among different hospitals; =0 | 0 |  |  |  |
| 7. Birth weight is necessary for DRGs grouping; =1 | 0.576 | 0.507 | -0.418—1.569 | 0.256 |
| 7. Birth weight is necessary for DRGs grouping; =0 | 0 |  |  |  |
| 8. Cases treated with costly, high-priced medicines are separately paid as special cases; =1 | -0.497 | 0.573 | -1.620—0.626 | 0.386 |
| 8. Cases treated with costly, high-priced medicines are separately paid as special cases; =0 | 0 |  |  |  |
| 9. Compared with the previous period, payment by DRGs is conducive to controlling the increase of medical expenses; =1 | 2.555 | 0.638 | 1.305—3.805 | < 0.001 |
| 9. Compared with the previous period, payment by DRGs is conducive to controlling the increase of medical expenses; =0 | 0 |  |  |  |
| 10. The implementation of actual payment by DRGs by the Beijing Municipal Health Insurance Bureau is on March 2022; =1 | 0.719 | 0.642 | -0.540—1.978 | 0.263 |
| 10. The implementation of actual payment by DRGs by the Beijing Municipal Health Insurance Bureau is on March 2022; =0 | 0 |  |  |  |

Supplementary table 2 Parameter estimates of attitudes effects on practices

| Parameters (Attitude item) | Coefficient | Standard error | 95% Wald CI | P value |
| --- | --- | --- | --- | --- |
| Intercept | 14.584 | 2.443 | 9.795—19.373 | 0.000 |
| 1. Compared to the previous period, medical staff are able to help patients more effectively after payment by DRGs; =5 | 0.647 | 0.565 | -0.461—1.755 | 0.252 |
| 1. Compared to the previous period, medical staff are able to help patients more effectively after payment by DRGs; =4 | 0.688 | 0.524 | -0.339—1.715 | 0.189 |
| 1. Compared to the previous period, medical staff are able to help patients more effectively after payment by DRGs; =3 | 0.490 | 0.506 | -0.503—1.482 | 0.333 |
| 1. Compared to the previous period, medical staff are able to help patients more effectively after payment by DRGs; =2 | 0.830 | 0.495 | -0.140—1.800 | 0.093 |
| 1. Compared to the previous period, medical staff are able to help patients more effectively after payment by DRGs; =1 | 0 |  |  |  |
| 2. I have had to deal with more administrative issues in order to adapt to the implementation of payment by DRGs; =5 | 1.456 | 1.190 | -0.87—3.789 | 0.221 |
| 2. I have had to deal with more administrative issues in order to adapt to the implementation of payment by DRGs; =4 | 1.380 | 1.201 | -0.974—3.735 | 0.251 |
| 2. I have had to deal with more administrative issues in order to adapt to the implementation of payment by DRGs; =3 | 1.336 | 1.176 | -0.967—3.640 | 0.256 |
| 2. I have had to deal with more administrative issues in order to adapt to the implementation of payment by DRGs; =2 | 0.637 | 1.313 | -1.936—3.210 | 0.627 |
| 2. I have had to deal with more administrative issues in order to adapt to the implementation of payment by DRGs; =1 | 0 |  |  |  |
| 3. Payment by DRGs system will possibly increase my personal income; =5 | -0.265 | 0.917 | -2.062—1.532 | 0.772 |
| 3. Payment by DRGs system will possibly increase my personal income; =4 | -3.071 | 0.577 | -4.202—-1.940 | 0.000 |
| 3. Payment by DRGs system will possibly increase my personal income; =3 | -0.366 | 0.321 | -0.996—0.264 | 0.255 |
| 3. Payment by DRGs system will possibly increase my personal income; =2 | -0.434 | 0.331 | -1.082—0.214 | 0.189 |
| 3. Payment by DRGs system will possibly increase my personal income; =1 | 0 |  |  |  |
| 4. Grouping by DRGs will force clinicians to focus more on the cost of patient therapy; =5 | 2.037 | 1.959 | -1.802—5.876 | 0.298 |
| 4. Grouping by DRGs will force clinicians to focus more on the cost of patient therapy; =4 | 2.011 | 1.965 | -1.840—5.862 | 0.306 |
| 4. Grouping by DRGs will force clinicians to focus more on the cost of patient therapy; =3 | 1.941 | 2.000 | -1.979—5.862 | 0.332 |
| 4. Grouping by DRGs will force clinicians to focus more on the cost of patient therapy; =2 | 3.266 | 2.112 | -0.873—7.405 | 0.122 |
| 4. Grouping by DRGs will force clinicians to focus more on the cost of patient therapy; =1 | 0 |  |  |  |
| 5. Payment by DRGs groups hospitals to declare a faster return of health insurance funds; =5 | 0.704 | 0.841 | -0.945—2.352 | 0.403 |
| 5. Payment by DRGs groups hospitals to declare a faster return of health insurance funds; =4 | 0.627 | 0.767 | -0.876—2.131 | 0.413 |
| 5. Payment by DRGs groups hospitals to declare a faster return of health insurance funds; =3 | 0.482 | 0.705 | -0.901—1.864 | 0.495 |
| 5. Payment by DRGs groups hospitals to declare a faster return of health insurance funds; =2 | -0.349 | 0.910 | -2.132—1.434 | 0.701 |
| 5. Payment by DRGs groups hospitals to declare a faster return of health insurance funds; =1 | 0 |  |  |  |
| 6. Clinicians should invest more effort in achieving clinical pathways; =5 | -0.077 | 1.180 | -2.391—2.236 | 0.948 |
| 6. Clinicians should invest more effort in achieving clinical pathways; =4 | 0.458 | 1.191 | -1.877—2.792 | 0.701 |
| 6. Clinicians should invest more effort in achieving clinical pathways; =3 | -0.888 | 1.236 | -3.310—1.533 | 0.472 |
| 6. Clinicians should invest more effort in achieving clinical pathways; =2 | -0.251 | 1.255 | -2.711—2.209 | 0.841 |
| 6. Clinicians should invest more effort in achieving clinical pathways; =1 | 0 |  |  |  |
| 7. The hospital I work at has made good cost measurements for the DRGs-based payment system; =5 | -1.715 | 1.556 | -4.765—1.336 | 0.271 |
| 7. The hospital I work at has made good cost measurements for the DRGs-based payment system; =4 | -2.088 | 1.562 | -5.150—0.974 | 0.181 |
| 7. The hospital I work at has made good cost measurements for the DRGs-based payment system; =3 | -2.505 | 1.564 | -5.571—0.560 | 0.109 |
| 7. The hospital I work at has made good cost measurements for the DRGs-based payment system; =2 | -3.024 | 2.133 | -7.204—1.156 | 0.156 |
| 7. The hospital I work at has made good cost measurements for the DRGs-based payment system; =1 | 0 |  |  |  |
| 8. Payment system based on DRGs will probably restrain physicians' career development; =5 | 0.837 | 1.265 | -1.643—3.316 | 0.508 |
| 8. Payment system based on DRGs will probably restrain physicians' career development; =4 | -0.022 | 0.518 | -1.038—0.994 | 0.966 |
| 8. Payment system based on DRGs will probably restrain physicians' career development; =3 | -0.572 | 0.429 | -1.412—0.269 | 0.182 |
| 8. Payment system based on DRGs will probably restrain physicians' career development; =2 | -0.225 | 0.398 | -1.005—0.554 | 0.571 |
| 8. Payment system based on DRGs will probably restrain physicians' career development; =1 | 0 |  |  |  |
| 9. Payment system based on DRGs is not favorable to the use of medical innovations; =5 | -0.056 | 0.683 | -1.395—1.283 | 0.935 |
| 9. Payment system based on DRGs is not favorable to the use of medical innovations; =4 | 0.444 | 0.536 | -0.607—1.495 | 0.408 |
| 9. Payment system based on DRGs is not favorable to the use of medical innovations; =3 | -0.119 | 0.387 | -0.877—0.639 | 0.758 |
| 9. Payment system based on DRGs is not favorable to the use of medical innovations; =2 | 0.025 | 0.315 | -0.592—0.643 | 0.936 |
| 9. Payment system based on DRGs is not favorable to the use of medical innovations; =1 | 0 |  |  |  |
